# Crizotinib Exhibits Antitumor Activity by Targeting ALK Signaling not c-MET in Pancreatic Cancer

**DOI:** 10.18632/oncotarget.2363

**Published:** 2014-08-23

**Authors:** Hong Hua Yan, Kyung Hee Jung, Mi Kwon Son, Zhenghuan Fang, Soo Jung Kim, Ye-Lim Ryu, Juyoung Kim, Mi-Hyun Kim, Soon-Sun Hong

**Affiliations:** ^1^ College of Medicine, Inha University, 3-ga, Sinheung-dong, Jung-gu, Incheon 400-712, Republic of Korea;; ^2^ School of Biological & Chemical Engineering, Yanbian University of Science & Technology, Beishan St., Yanji City, Jilin Prov., 133000, China

**Keywords:** Crizotinib, Pancreatic cancer, c-MET, ALK, Apoptosis

## Abstract

Crizotinib, a c-MET/ALK inhibitor, has exhibited antitumor efficacy in different types of cancers. However, studies regarding Crizotinib in pancreatic cancer have been limited. Thus, we investigated the effect of Crizotinib on pancreatic cancer and its mechanism of action. Crizotinib strongly suppressed the growth and proliferation of pancreatic cancer cells in a dose-dependent manner. Also, it induced apoptosis by modulating its related factors. In the study, with regard to the mechanism of action, Crizotinib did not inhibit c-MET expression on pancreatic cancer cells; instead, it specifically inhibited the activity of ALK, which was identified to be highly expressed on various pancreatic cancer cells and tissues in our study. In 42 different receptor tyrosine kinase (RTKs) array, Crizotinib also strongly inhibited the expression of activated ALK in pancreatic cancer cells, modulating its downstream mediators such as STAT3, AKT, and ERK. Furthermore, Crizotinib inhibited angiogenesis in a mouse Matrigel plug assay as well as the progression of tumor growth in a mouse xenograft model. Taken together, our investigation shows that Crizotinib inhibits the ALK signaling pathway in pancreatic cancer, resulting in cell growth/angiogenesis inhibition and apoptosis induction. We suggest that Crizotinib might be used as a novel therapeutic drug for treating pancreatic cancer.

## INTRODUCTION

Advanced stage pancreatic cancer is the fourth leading cause of cancer-related deaths in the United State [1]. Even though extensive efforts have been made to treat pancreatic cancer, its aggressive behavior and intrinsic resistance to most chemotherapeutic agents lead to less than a 5% overall 5-year survival rate [2]. There are only a small percent of selected patients who are able to undergo a surgical operation. To date, several regimens are widely used to treat advanced pancreatic cancer, including gemcitabine alone or in combination with other drugs; however, it is disappointing that there is no clearly superior treatment option for locally advanced pancreatic cancer [3-5].

The mesenchymal–epithelial transition factor (c-MET) is one of the subfamilies of receptor tyrosine kinase (RTKs) for hepatocyte growth factor (HGF), which functions to mediate cell proliferation, invasion, motility and survival. During embryogenesis and tissue repair, c-MET signaling is critical for normal process [6]. It has been reported that c-MET was highly expressed in many types of human cancers, such as liver, ovarian, non-small cell lung, gastric cancers, and pancreatic cancer [7]. Also, c-MET was frequently overexpressed, mutated or amplified in many cancers. In pancreatic cancer, c-MET was overexpressed up to 80% of pancreatic ductal adenocarcinoma cases, and c-MET alterations were shown to be a strong indicator for increased recurrence rates and overall poor survival for pancreatic ductal adenocarcinoma patient [8-11]. Recently, c-MET has been identified as a new marker for pancreatic stem cells and therapeutic target in pancreatic cancer. In addition, c-MET inhibitor, such as Cabozantinib, has overcome gemcitabine resistance in pancreatic cancer cells [12, 13].

Anaplastic lymphoma kinase (ALK) is one of the insulin receptor superfamily of RTKs, and it was firstly identified as a result of cloning the nuclear protein nucleophosmin (NPM)–ALK fusion gene in anaplastic large-cell lymphomas [14]. ALK pathway is one of the most frequently deregulated pathways in various human cancers [15]. Activation of ALK mediates many functional processes, including cell proliferation, survival, division, and invasion through the modulation of signaling pathways, such as PI3K/AKT, STAT3 and RAS/MEK [16, 17]. Translocation of ALK was reported as the most common cause of genomic ALK aberration in many cancers, including anaplastic large-cell lymphomas, non-small cell lung cancer, diffuse large B-cell lymphoma, and inflammatory myofibroblastic tumor. In addition, ALK was also mutated or amplified frequently in many cancers. For this reason, targeting ALK signaling has emerged as an attractive therapy in many types of ALK alteration cancers [18, 19].

Crizotinib is an ATP-competitive small molecule and orally bioavailable dual inhibitor of the c-MET/HGF receptor and ALK tyrosine kinases [20]. In early studies, Crizotinib inhibited tumor cell proliferation and microvessel density, and also induced apoptosis in many types of cancers, including gastric cancer, non-small cell lung cancer, and ovarian cancer. In clinical trials, Crizotinib was approved by the US Food and Drug Administration (FDA) as a treatment for locally advanced or metastatic ALK-rearranged non-small cell lung cancer in 2011. This approval was dependent on response rates of 50 - 61% from 255 ALK-rearranged non-small cell lung cancer patients enrolled in two single-arm trials [21, 22].

Although physiological and anticancer effects of Crizotinib were documented in many cancers, anticancer activities of Crizotinib in pancreatic cancer have not been fully investigated [16]. Therefore, we investigated the anticancer effect of Crizotinib and its mechanism of action in pancreatic cancer. Our present study revealed that Crizotinib induced apoptosis and inhibited cell growth or angiogenesis via inhibition of ALK signaling, not c-MET signaling in pancreatic cancer.

## RESULTS

### Crizotinib inhibited cell growth and proliferation in pancreatic cancer cells

**Figure 1:**
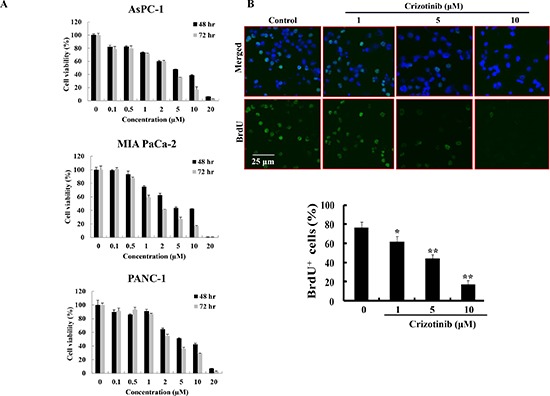
Effect of Crizotinib on the proliferation of human pancreatic cancer cells **(A)** Cell viability of Crizotinib was measured by MTT assay in AsPC-1, PANC-1, and MIA PaCa-2 cells at 48 hr or 72 hr. **(B)** BrdU staining was performed after PANC-1 cells were treated with or without various concentrations of Crizotinib for 6 hr (*p < 0.05 and **p < 0.01, compared to control). The upper panel represents a treatment with various concentrations of Crizotinib. The below panel shows the percent of BrdU-positive cells in several random fields of the BrdU staining images. All results are expressed as a percent cell proliferation relative to the control.

To evaluate the effect of Crizotinib on the growth and proliferation of pancreatic cancer cells (AsPC-1, PANC-1 and MIA PaCa-2), we used MTT assay and BrdU staining. The results from an MTT assay showed that Crizotinib inhibited the growth of pancreatic cancer cells in a dose-dependent manner for 48 hr and 72 hr with IC_50_ values close to 5 μM in the pancreatic cell lines (Fig. 1A). Using BrdU staining for 6 hr, we also found that Crizotinib inhibited the proliferation of PANC-1 cells in a dose-dependent manner (Fig. 1B).

### Crizotinib induced apoptosis in pancreatic cancer cells

**Figure 2:**
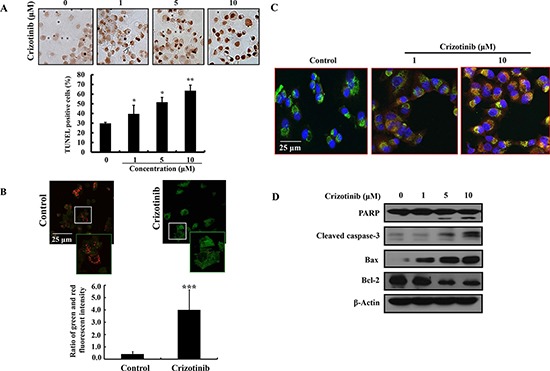
Induction of apoptosis by Crizotinib treatment in PANC-1 pancreatic cancer cells **(A)** The induction of apoptosis by Crizotinib was confirmed by TUNEL assay. (*p < 0.05 and **p < 0.01, compared to control). **(B)** JC-1 staining was performed to detect the mitochondrial depolarization that occurs in apoptosis after a treatment with 10 μM of Crizotinib for 6 hr. Depolarized regions are indicated by the green fluorescence. (400x magnification) (***p < 0.001, compared to control). **(C)** PANC-1 pancreatic cancer cells were treated with Crizotinib (1 and 10 μM) for 6 hr and stained with anti-cytochrome c antibody, Mitotracker and DAPI. The immunostained cells were analyzed under an Olympus confocal laser scanning microscope with 400x magnification. **(D)** The expression of PARP, cleaved caspase-3, Bax, and Bcl-2 were measured by western blotting after PANC-1 cells were treated with various concentrations of Crizotinib (0-10 μM) for 72 hr.

To further know whether Crizotinib induces apoptosis in pancreatic cancer cells, we performed TUNEL assay, JC-1 staining, MitoTracker probe, and western blotting in PANC-1 cells. As observed by TUNEL staining, DNA fragments were detected after Crizotinib treatment (Fig. 2A). During apoptosis, several key events happen in the mitochondria, including the release of cytochrome *c*, changes in electron transport, and loss of mitochondrial transmembrane potential. As shown in Fig. 2B, mitochondrial transmembrane potential was changed markedly as evident from the disappearance of red fluorescence or the increase of green fluorescence in most cells. Consistent with the above results, the release of cytochrome *c* into the cytoplasm was induced by the change of mitochondrial transmembrane potential as shown in Fig. 2C. In addition, Crizotinib increased the expression levels of cleaved caspase-3 and PARP, as well as the Bax expression. In contrast, the expression level of Bcl-2 was decreased by Crizotinib.

### Crizotinib did not inhibit the phosphorylation of c-MET in pancreatic cancer cells

**Figure 3:**
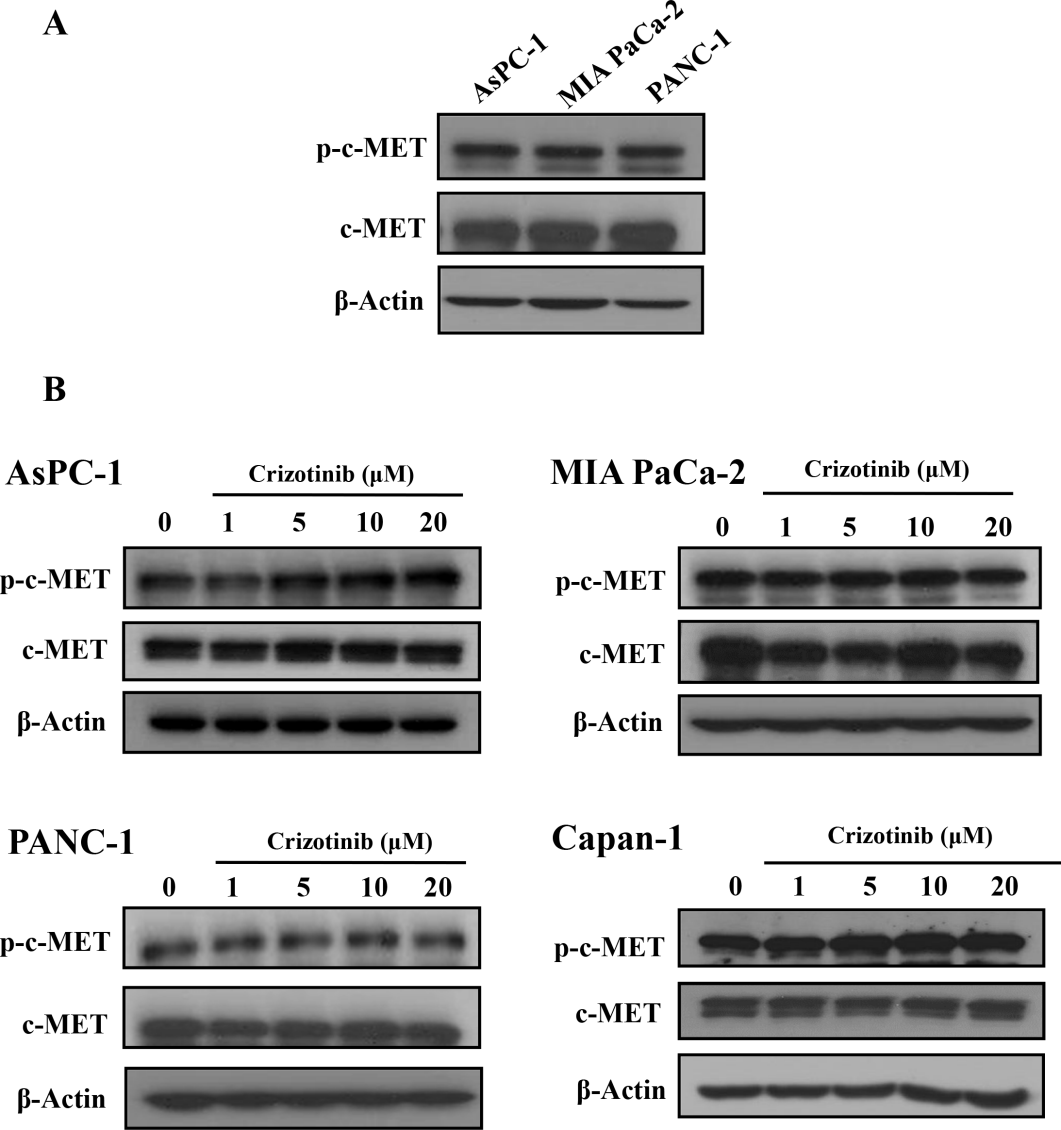
Effect of Crizotinib on c-MET expression in pancreatic cancer cells **(A)** Expression of c-MET and p-c-MET on pancreatic cancer cells were determined by Western blotting. **(B)** Pancreatic cancer cells were treated with Crizotinib in various concentrations (1-20 μM).

It has been reported that c-MET was highly expressed in a variety of carcinomas, including lung cancer, breast cancer, colon cancer, and pancreatic cancer [7]. c-MET inhibitors, including Crizotinib and Cabozantinib, enhanced the antitumor effect of gemcitabine in pancreatic cancers. Also, Crizotinib as a c-MET inhibitor showed an antitumor effect via inhibition of c-MET signaling in lung and gastric cancer cells [13, 23, 24]. Hence, we identified the expression of c-MET in pancreatic cancer and then investigated whether Crizotinib inhibited the phosphorylation of c-MET in pancreatic cancer cells. As shown in Fig. 3A, c-MET and p-c-MET were highly expressed in pancreatic cancer cell lines. When pancreatic cancer cells were treated with Crizotinib in a dose-dependent manner, it did not inhibit the expression of both p-c-MET and c-MET (Fig. 3B).

### Crizotinib inhibited the phosphorylation of c-MET in c-MET amplification cells, not in c-MET overexpression or splice mutation cells

**Figure 4:**
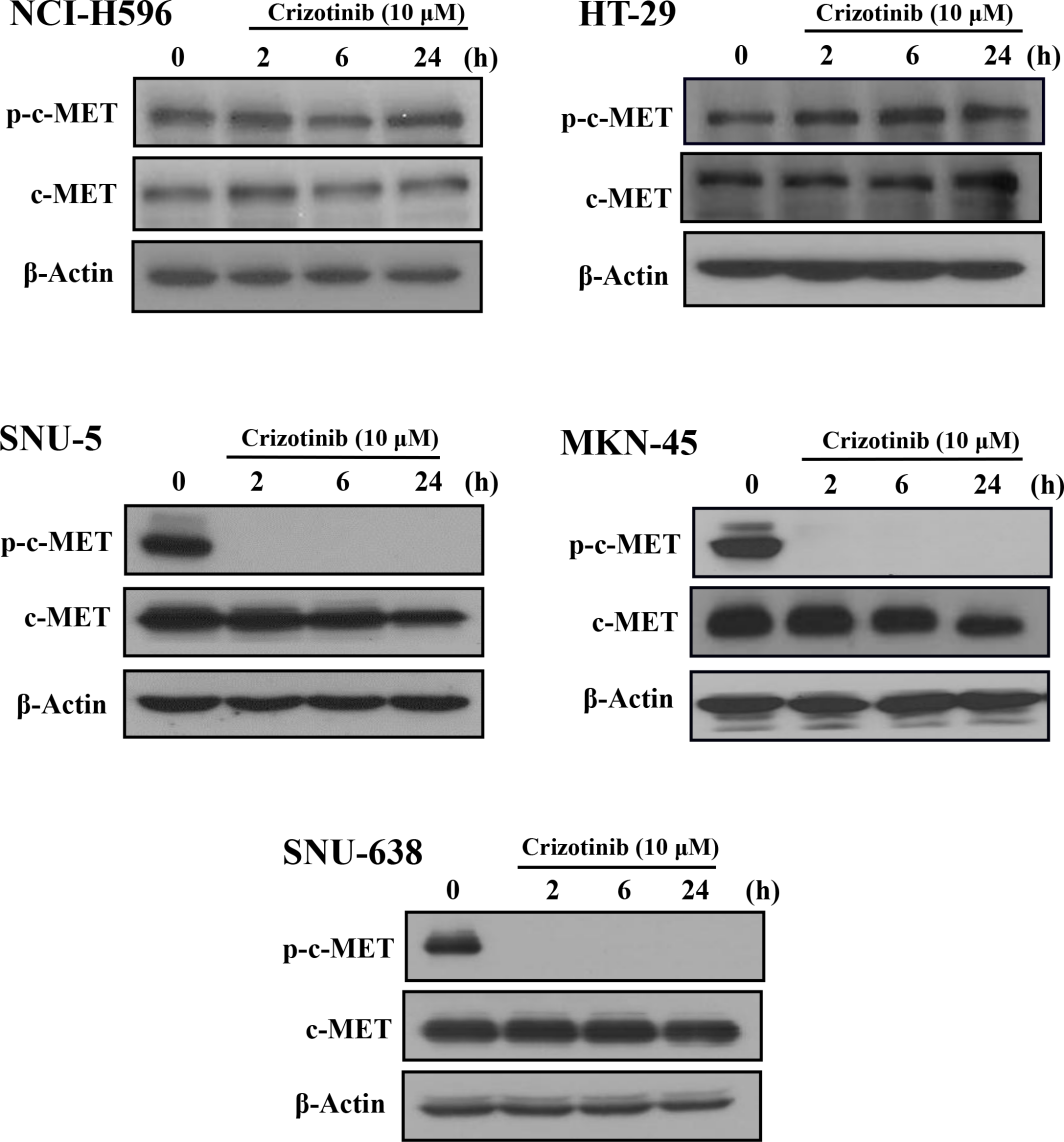
Effect of Crizotinib on c-MET expression in c-MET altered cancer cells Cancer cells with c-MET alterations were exposed to Crizotinib (10 μM) in the indicated times (SNU-5, MKN-45, and SNU-638: gastric cancer cells of c-MET amplification; NCI-H596: non-small cell lung cancer cells of c-MET splicing mutation; HT-29: colon cancer cells of c-MET overexpression).

Some reports have demonstrated that Crizotinib inhibited the proliferation and growth by inhibiting c-MET signaling in c-MET altered cancers [24]. However, in this study, Crizotinib did not inhibit the phosphorylation of c-MET in pancreatic cancer cells. To further speculate the reason, we used three types of c-MET altered cancer cell lines including SNU-5, MKN-45, and SNU-638 gastric cancer cells (c-MET amplification), NCI-H596 non-small cell lung cancer (c-MET splice mutation) and HT-29 colon cancer cells (c-MET overexpression). As shown in Fig. 4, Crizotinib (10 μM) obviously inhibited the phosphorylation of c-MET in SNU-5 cells, MKN-45, and SNU-638 cells; while the phosphorylation of c-MET was not inhibited in HT-29 and NCI-H596 cells. According to the result, Crizotinib inhibited the phosphorylation of c-MET only in c-MET amplification cancer cells, and not in other types of c-MET alternated cancer cells.

### Crizotinib inhibited the phosphorylation of ALK in pancreatic cancer cells

**Figure 5:**
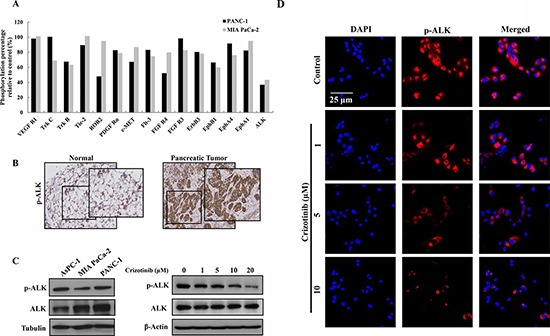
Expression of ALK in pancreatic cancer cells and tumors **(A)** After PANC-1 and MIA PaCa-2 cells were treated with Crizotinib (5 μM) for 2 hr, the phosphorylation level of receptor tyrosine kinase was analyzed by a Phospho-RTK assay. **(B)** ALK expression was determined in the human pancreatic tumor tissue. **(C)** ALK expression was determined in human pancreatic cancer cells. After PANC-1 cells were treated with various concentrations (0-20 μM) of Crizotinib for 2 hr, the expression of p-ALK was analyzed by Western blotting. **(D)** PANC-1 cells were also treated with various concentrations (0-10 μM) of Crizotinib and stained with p-ALK antibody and DAPI. The immunostained cells were analyzed under an Olympus confocal laser scanning microscope with 400x magnification.

To test which receptor tyrosine kinase (RTK) was regulated by Crizotinib, we performed a human Phospho-RTK array. 5 μM of Crizotinib was treated to PANC-1 and MIA PaCa-2 cells for 2 hr. As shown in Fig. 5A, Crizotinib decreased ALK phosphorylation more than any other RTKs, including c-MET. ALK expression has been found in several types of cancers, such as anaplastic large-cell lymphoma, non-small cell lung cancer, diffuse large B-cell lymphoma, and inflammatory myofibroblastic tumors [25]. However, studies regarding ALK expression in pancreatic cancer was limited. Therefore, we used tissue array to analyze the expression of p-ALK in human pancreatic tumor tissue. As shown in Fig. 5B, the expression of phosphorylated ALK was higher in pancreatic tumors than in the normal pancreas. Furthermore, p-ALK was significantly expressed in pancreatic cancer cell lines (AsPC-1, MIA PaCa-2, PANC-1) we used. When PANC-1 cells were exposed to Crizotinib for 6 hr, phosphorylation of ALK was reduced in a dose dependent manner (Fig. 5C and 5D).

### Crizotinib inhibited ALK downstream pathway in pancreatic cancer cells

**Figure 6:**
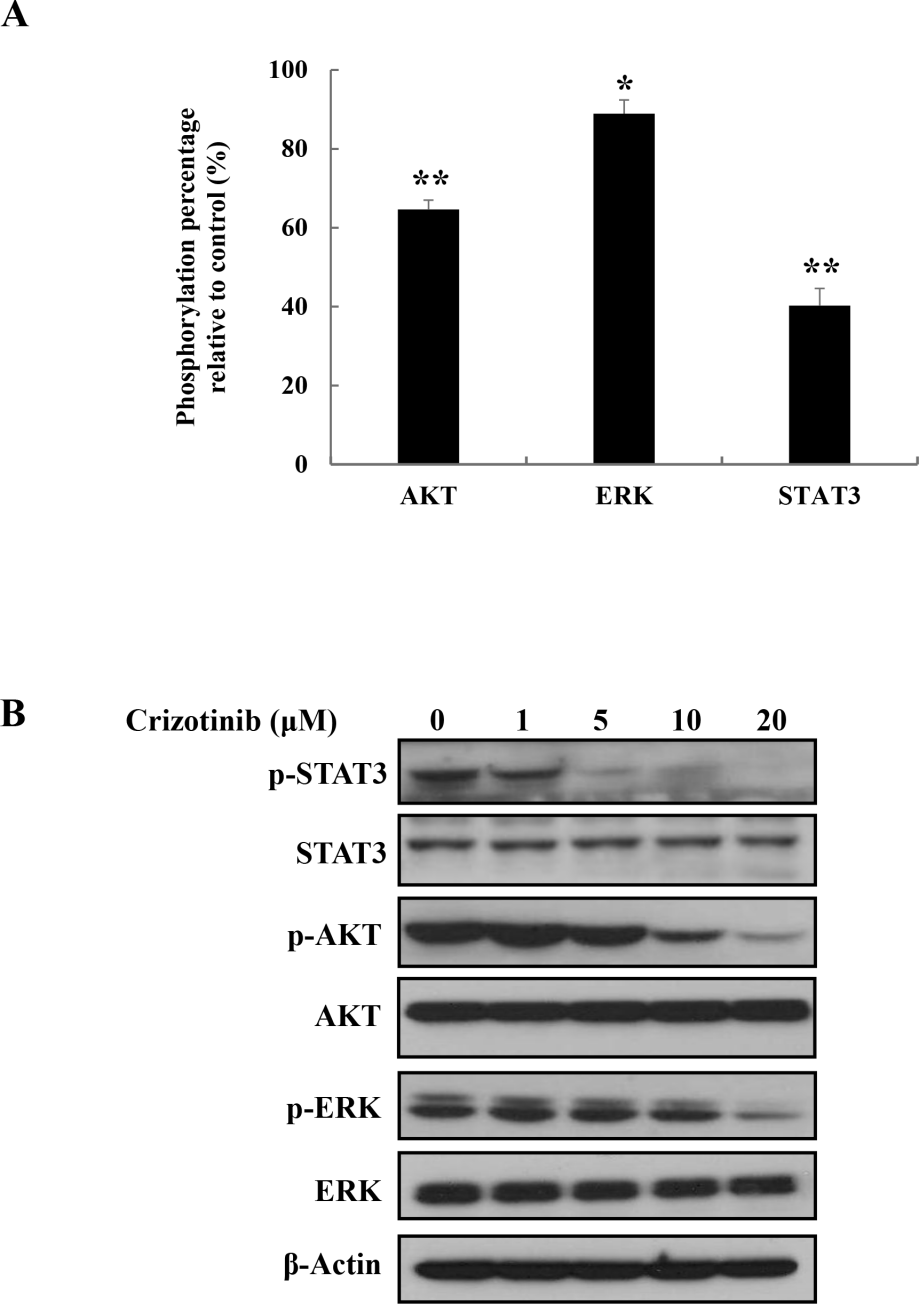
Inhibition of ALK signaling pathway by Crizotinib in PANC-1 cells **(A)** The ability of Crizotinib to target ALK signaling pathway was analyzed by Phospho-Kinase array after PANC-1 cells were treated with Crizotinib (5 μM) for 2 hr (*p < 0.05 and ** p <0.01, compared to control). **(B)** The expression of p-STAT3, p-AKT, and p-ERK were measured by western blotting after PANC-1 cells were treated with Crizotinib at the indicated doses (0-20 μM) for 2 hr.

To evaluate the ability of Crizotinib to target downstream signaling pathways, we detected phosphorylation level of downstream proteins by using human Phospho-Kinase assay and western blotting. When PANC-1 cells were treated with 5 μM of Crizotinib for 2 hr, we observed that Crizotinib down-regulated JAK/STAT3, PI3K/AKT, and MEK/ERK1/2 signaling pathways, which are known as the main ALK downstream signaling pathway [18] (Fig. 6A). In particular, JAK/STAT3 pathway was markedly inhibited by Crizotinib. These results were also confirmed by western blotting. Indeed, Crizotinib effectively suppressed the downstream factors, such as STAT3, AKT, and ERK in a dose dependent manner.

### Crizotinib inhibited tumor growth in PANC-1 pancreatic cancer xenograft models

**Figure 7:**
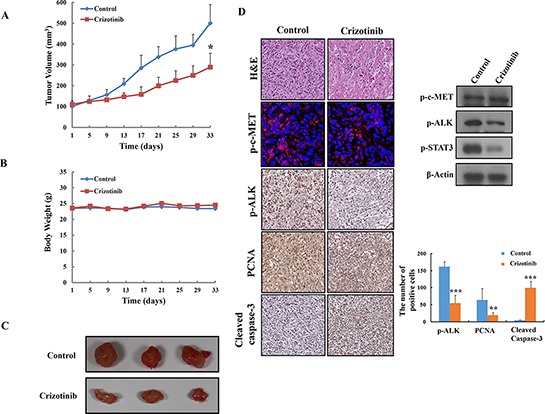
Inhibition of tumor growth by Crizotinib in PANC-1 pancreatic cancer xenograft models **(A)** Effect of Crizotinib on tumor size in PANC-1 pancreatic cancer xenograft models (n=5). The volume of each tumor was measured every 3 days. The average tumor volume in the vehicle or Crizotinib (50 mg/kg) treated group was plotted (* p <0.05, compared to control). **(B)** Effect of Crizotinib on body weight change. The weight of each mouse was measured every 3 days. **(C)** The size of isolated tumor from PANC-1 xenograft models. The results are expressed as the mean ± S. D. (n = 5). **(D)** H&E staining, expression of p-c-MET, p-ALK, PCNA, Cleaved caspase-3, and p-STAT3 was confirmed by immunohistochemistry, immunofluoroscence, and western blotting in pancreatic tumors. The expression of p-ALK, PCNA, cleaved caspase-3 were quantified (**p < 0.01 and *** p <0.001, compared to control).

Based on the above *in vitro* findings, we extended our study to an *in vivo* xenograft model. After inoculation with PANC-1 pancreatic cancer cells, mice were administrated by oral gavage with Crizotinib at a dose of 50 mg/kg for 33 days (n=5, each group). Tumor volume was significantly suppressed in the Crizotinib (50 mg/kg) treated groups (*p < 0.05, Fig. 7A and 7C). Moreover, the Crizotinib treated group did not show any body weight change at the tested dosage (Fig. 7B), which indicates little toxicity of the compound. In histopathological analysis, we observed that there was a greater degree of tumor apoptosis in the Crizotinib treated group compared with the control group.

Next, immunohistochemistry and western blotting was performed to identify the expression of PCNA (a representative marker of proliferation), c-MET, p-ALK, and cleaved caspase-3, and p-STAT3. As shown in Fig. 7D, the Crizotinib treated group resulted in significant decreased PCNA, and p-ALK, p-STAT3 and compared with the control group. Also, the apoptotic effect of Crizotinib on pancreatic tumor tissues was observed in the increased expression of cleaved caspase-3. However, we observed that Crizotinib did not decrease expression of p-c-MET in tumor tissue using immunostaining and western blotting.

### Crizotinib inhibited angiogenesis in the Matrigel plug assay

**Figure 8:**
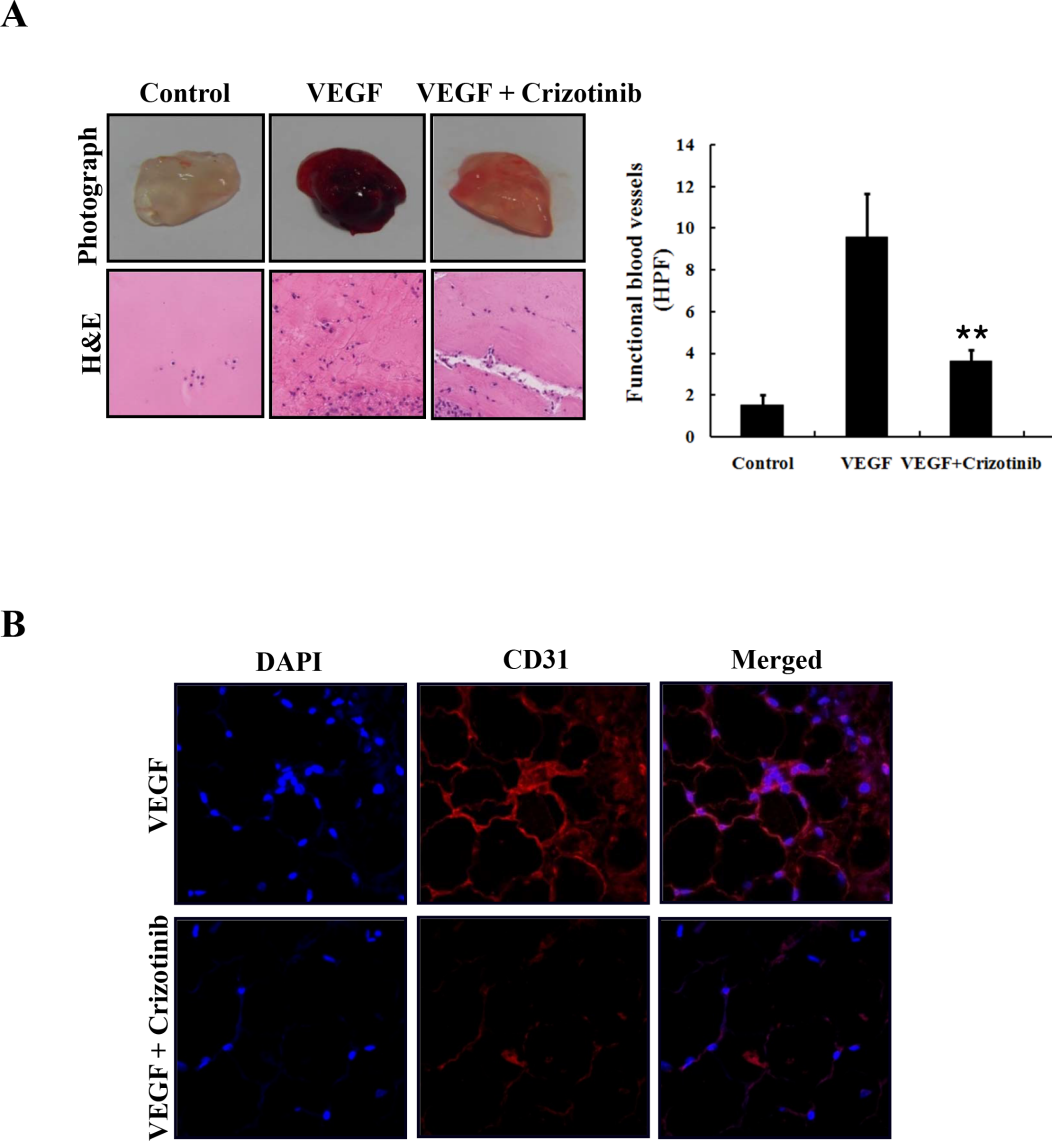
Inhibition of angiogenesis by Crizotinib **(A)** Matrigel plugs containing VEGF (50 ng/mL) and/or Crizotinib (5 μM) were implanted into the mice and then evaluated for vascularization after 7 days. The plugs were sectioned and stained with H&E. The infiltrating microvessels with intact RBC were quantified (**p < 0.01, compared to VEGF). **(B)** Endothelial cells in the plug were immunostained with CD31, and the stained plug was observed by microscopy with 400x magnification.

In order to identify whether Crizotinib inhibits *in vivo* vascularization, we performed a Matrigel plug assay. As shown in Fig. 8A, blood vessels were rarely observed in the Matrigel plugs without VEGF. In contrast, neovessel containing intact red blood cells, inside the Matrigel, were induced by VEGF, which was obviously inhibited in the presence of 5 μM of Crizotinib. For a histological analysis, each section of the Matrigel plug was stained with H&E and endothelial marker CD31, respectively. The results showed that the plug with Crizotinib treatment had fewer vessels than those induced by VEGF. CD31 expression was also decreased by Crizotinib treatment in the VEGF-induced Matrigel plug. These results suggest that Crizotinib possessed a potent anti-angiogenic activity *in vivo* (Fig. 8B).

## DISCUSSION

Current therapeutic approaches for patients with unresectable pancreatic cancer are chemoraidotherapy or chemotherapy alone. Although gemcitabine was adopted as the standard therapy of advanced pancreatic cancer, it has shown to have a low-response rate and a fast resistance development. Also, combination therapy, such as FOLFIRINOX (5-fluorouracil, leucovorin, irinotecan and oxaliplatin), extended life by only 4 months compared with gemcitabine [26]. In addition, this combination therapy presented severe side effects in pancreatic cancer patients. For this reason, the targeted therapies have generated a lot of interest in discovering better approaches for patients with pancreatic cancer. Crizotinib was initially developed as a c-MET inhibitor [7, 27]. A few years ago, Crizotinib has been extensively validated as a highly specific inhibitor of not only c-MET, but also ALK among > 120 different RTKs surveyed [28]. Based on previous studies, we set out to evaluate the anticancer effects of Crizotinib and its mechanism of action in pancreatic cancer. For the first time, we report that Crizotinib inhibited ALK signaling pathway and not c-MET, which may lead to the induction of apoptosis along with the inhibition of cell growth and angiogenesis in pancreatic cancer.

Crizotinib has exhibited anticancer effects in several types of cancers, including gastric cancer and non-small cell lung cancer [29, 30]. However, there are few studies for anticancer effect of Crizotinib in pancreatic cancer. In this study, we observed that Crizotinib strongly suppressed cell growth and DNA synthesis of the three pancreatic cancer cells. Also, Crizotinib had a strong anticancer effect by up-regulating various apoptosis related molecules in pancreatic cancer cells. These results were also confirmed by *in vivo* data that Crizotinib inhibited tumor growth in xenograft animal models.

Recent studies have reported that c-MET was considered as a potential target in pancreatic cancer [7, 31]. Therefore, we assumed that the anticancer effect of Crizotinib (c-MET/ALK inhibitor) in this study would be induced by targeting c-MET. However, Crizotinib did not inhibit the expression of p-c-MET in pancreatic cancer cells. It seems because Crizotinib did not function in pancreatic cancer with overexpressed c-MET [8, 32]. Indeed, Okamato *et al.* reported that Crizotinib specifically showed antitumor activities in gastric cancer with c-MET amplification [29]. To confirm these observations, we identified the activation of c-MET by Crizotinib in various c-MET altered cells, including SNU-5, MKN-45, and SNU-638 cells (c-MET amplification), NCI-H596 (c-MET splicing mutation), and HT-29 cells (c-MET overexpression). Interestingly, Crizotinib inhibited activation of c-MET only in c-MET amplified SNU cells, and not in NCI-H596 (c-MET splicing mutation) or HT-29 cells (c-MET overexpression). This is consistent with the studies of Tanizaki *et al.* and Hong *et al.,* where Crizotinib showed a marked anticancer action in c-MET amplification-positive cancer cells, but not in c-MET mutated cells [32, 33].

To investigate the possible target mechanism involved in anticancer activities of Crizotinib, we used a RTK array. Interestingly, we found that among the 42 RTKs, Crizotinib inhibited the phosphorylation of ALK more than other tyrosine kinase receptors in both PANC-1 and MIA PaCa-2 pancreatic cancer cells. In addition, to observe the expression of ALK in pancreatic cancer patients, we performed a human tissue array. Surprisingly, ALK was highly expressed in pancreatic cancer cells than normal pancreas tissues. Further, in pancreatic cancer cell lines used in this study, high expression of ALK was observed (AsPC-1, MIA PaCa-2, PANC-1). In previous studies, ALK was involved in tumor progression of several cancers, including anaplastic large cell lymphomas, ALK-positive diffuse large B-cell lymphomas, inflammatory myofibroblastic tumors, and non-small cell lung carcinomas [15]. Given the fact that ALK plays an important role in tumorigenesis as expressing high levels in tumors, whereas the normal ALK protein is absent or present at low levels in normal cells, targeting ALK is expected to be an attractive anticancer therapy [19]. Up until now, Crizotinib has been considered as the first-generation ALK inhibitor and have inhibited the activity of ALK in various cancers with ALK alteration [19, 27]. Therefore, we investigated the effect of Crizotinib on the expression of p-ALK in pancreatic cancer. From our results, Crizotinib inhibited the expression of p-ALK in PANC-1 pancreatic cancer cells, in a dose dependent manner. Although relevance between the expression of ALK and ALK gene alteration in pancreatic cancer remains to be further investigated, Crizotinib obviously showed an anticancer effect via target inhibition of ALK, and not c-MET.

A lot of studies have indicated that ALK activated various pathways including the JAK/STAT3 pathway, PI3K/AKT pathway, and RAS/RAF/MEK/ERK1/2 pathway, which control cell proliferation and survival [16, 19]. Therefore, to evaluate the ability of Crizotinib in targeting downstream signaling pathways, we used a Phospho-Kinase array. Among those downstream signaling pathways, Crizotinib effectively inhibited the phosphorylation of STAT3 in PANC-1 cells. Furthermore, Crizotinib slightly affected the phosphorylation of AKT and ERK (one of the MAPKs), the main mediators of the ALK signaling pathway [16, 18]. These data indicate that Crizotinib may inhibit the ALK/STAT3 pathway. Overall, we suggest that the anticancer effect of Crizotinib is associated with the inhibition of ALK signaling pathway, which finally leads to the inhibition of tumor growth and induction of apoptosis in PANC-1 xenograft models (Fig. 7).

Another anticancer effect of Crizotinib might be the suppression of angiogenesis. Angiogenesis is the process of new blood vessels sprouting from pre-existing structures, which occurs in the growth and progression of solid tumors, including pancreatic cancer [34, 35]. In addition, targeting angiogenesis provides the novel prognostic and therapeutic approaches to pancreatic cancer. Recently, antitumor study of Crizotinib has showed that microvessel density was reduced in a dose-dependent manner (CD31) [22]. Consistent with their results, our study showed that Crizotinib obviously inhibited the formation of new blood vessels in Matrigel plug assay, as well as decreasing the expression of CD31, suggesting that Crizotinib is a potential anti-angiogenic agent.

In conclusion, we demonstrate that Crizotinib suppresses tumor growth and angiogenesis and induces apoptosis by downregulating the ALK signaling pathway, and not c-MET in pancreatic cancer. Our discovery of this novel anticancer mechanism of Crizotinib may be useful for a novel drug targeting ALK in pancreatic cancer.

## MARERIALS AND METHODS

### Cells and materials

Human pancreatic cancer cell lines AsPC-1, PANC-1, MIA PaCa-2, and Capan-1 were purchased from the American Type Culture Collection (ATCC Manassas, VA). PANC-1 and MIA PaCa-2 were cultured in DMEM medium, Capan-1 was cultured in IMDM, and AsPC-1 was cultured in RPMI 1640 medium supplemented with 10% FBS and 1% penicillin/ streptomycin. Cell culture media, FBS, penicillin-streptomycin, and other supplementary agents were purchased from GIBCO. 3-(4,5-dimethylthiazol-2-yl)-2,5-diphenyltetrazolium bromide (MTT) was purchased from Sigma-Aldrich (St. Louis, MO). All cell lines were maintained in a CO_2_ incubator with a controlled humidified atmosphere composed of 95% air and 5% CO_2_. Crizotinib was purchased from LC laboratories (Woburn, MA) and dissolved in DMSO.

### Cell growth assay

AsPC-1, PANC-1, and MIA PaCa-2 were seeded 3-5×10^3^ cells/well in 96-well plates and incubated overnight for attachment, and Crizotinib was treated for 48 hr or 72 hr. After incubation, 20 μl of MTT solution (2 mg/mL) was added for 4 hr at 37°C, then the medium was removed. And 200 μl of DMSO was added to each well to dissolve the formazan crystals by constant shaking for 10 min. The plates were read at 540 nm on a microplate reader. MTT assay was repeated 3 times and a dose-response curve was used to assess IC_50_.

### 5′-bromo-2′-deoxyuridine (BrdU) cell proliferation assay

PANC-1 cells were plated onto an 18-mm cover glass in media and incubated overnight for attachment. The cells were exposed to various concentrations of Crizotinib and labeled with 10 μM of 5′-bromo-2′-deoxyuridine (BrdU) for 4 hr. The cells were then fixed in ice-cold ethanol and acetic acid mixture (2:1) for 5 min and washed with PBS (pH 7.4) containing 1% Triton X-100. Then, fixed cells were incubated with 4 N HCl to denature DNA for 15 min at 37°C and neutralized with 0.1 M sodium borate (pH 8.5). The cells were washed with 0.1% NP-40 in PBS for several times and blocked with blocking buffer for 30 min at room temperature. Finally, the FITC-anti-BrdU (abcam: Cambridge, MA) antibody, diluted with PBS buffer containing 0.1% Triton X-100, was used to measure the BrdU incorporation by a fluorescence microscopy.

### Terminal deoxynucleotidyl transferase-mediated nick end labeling (TUNEL) assay

PANC-1 cells were treated with various concentrations of Crizotinib (1-10 μM) for 24 hr. The cells were fixed in ice-cold 2% para-formaldehyde (PFA), washed with PBS, and terminal deoxynucleotidyl transferase-mediated nick end labeling (TUNEL) was subsequently performed using the TUNEL kit (Chemicon, Temecula, CA), following the manufacturer's instructions.

### Analysis of mitochondrial membrane potential

Mitochondrial membrane potential was measured using a mitochondria staining kit, called 5,5′,6,6′-tetrachloro -1,1′,3,3′-tetraethylbenzimidazol-carbocyanine iodide (JC-1: Grand Island, NY). Potential-dependent accumulation can be exhibited in the mitochondria. PANC-1 cells were plated on 18-mm cover glasses in DMEM medium and incubated for 24 hr. After attachment, the cells were treated with Crizotinib (10 μM) for 4 hr and added with 100 μ JC-1 solution (final concentration of 12.5 μg/ml) at 37°C for another 30 min. After washing the cells with PBS, they were stained with DAPI to visualize the nuclei. The cells were washed with PBS several times and covered with fluorescent mounting solution before viewing with a confocal laser scanning microscope (Olympus, Tokyo, Japan).

### Detection of Cytochrome c location

PANC-1 cells were treated with Crizotinib (1 μM and 10 μM) for 6 hr and then added with a mitochondrion-specific dye (MitoTracker red FM: Molecular probes Inc, Eugene, OR) for another 30 min at 37°C. The cells were washed with PBS and fixed in an acetone: methanol solution (1:2) for 10 min at −20°C. After washing with PBS for several times, the cells were incubated with cytochrome *c* antibody (1:50; Santa Cruz Biotechnologies, Santa Cruz, CA) overnight for 4°C. On the following day, the cells were again washed with PBS several times and incubated with a mouse fluorescence-labeled secondary antibody (1:100, Dianova, Hamburg, Germany) for 1 h at room temperature. The cells were stained with DAPI to visualize the nuclei. Finally, the cells were covered with a fluorescent mounting solution (Dako, Carpinteria, CA) before viewing with a confocal laser scanning microscope (Olympus).

### Western blotting

Total cellular proteins were extracted with a lysis buffer containing 1% Igepal CA-630, 20 mM Tris-HCl (pH 8.0), 137 μM NaCl, 10% glycerol, and 2 μM EDTA, as well as the following protease and phophatase inhibitor: aprotinin (10 mg/ml), leupeptin (10 mg/ml; ICN Biomedicals, Irvine, California), phenylmethylsulfonyl fluoride (1.72 μM), NaF (100 μM), NaVO_3_ (500 μM), and Na_4_P_2_O_7_ (500 mg/mL; Sigma-Aldrich). The proteins were separated by a sodium dodecylsulfate-polyacrylamide gel electrophoresis (SDS-PAGE), which were then transferred onto nitrocellulose membranes. The nitrocellulose membranes were immunostained with the appropriate primary antibodies, followed by the secondary antibodies conjugated to horseradish peroxidase. Antibody binding was detected with an enhanced chemiluminescence reagent (Amersham Biosciences, Buckinghamshire, UK). Antibodies against p-ALK(Tyr^1604^), ALK, p-c-MET (Tyr^1234/1235^), c-MET, p-ERK (Thr^202^/Tyr^204^), ERK, p-AKT (Ser^473^), AKT, p-STAT3 (Tyr^705^), STAT3, PARP, cleaved caspase-3, Bcl-2, Bax, and β-actin were purchased from Cell Signaling Technology (Danvers, MA) and abcam.

### Immunofluorescence

PANC-1 cells were treated with various (1-10 μM) concentrations of Crizotinib for 6 hr, washed with PBS twice, and fixed in acetone: methanol solution (1:1) for 10 min at −20°C. The fixed cells were washed and blocked in a blocking buffer for 1 hr at room temperature, and then incubated overnight with p-ALK antibody (1:50; Cell Signaling Technology) at 4°C. Before incubating with rabbit RITC secondary antibody (1:100, Dianova) for 1 hr at room temperature, the fixed cells were washed with PBS for several times. The fixed cells were also stained with 4,6-diamidino-2-phenylindole (DAPI) to visualize the nuclei. After washing with PBS twice, the slides were covered with DABCO (Sigma-Aldrich) and then viewed with a confocal laser scanning microscope (Olympus).

### Human Phospho-RTK assay

Human Phospho-RTK assay kit was purchased from R&D Systems (Minneapolis, MN). PANC-1 and MIA PaCa-2 cells were treated with Crizotinib (5 μM) for 2 hr. After blocking the membranes with a blocking buffer, the membranes were incubated with lysates extracting from PANC-1 and MIA PaCa-2 cells by using a lysis buffer overnight. The membranes were washed with a washing buffer 3 times, incubated with anti-Phospho-Tyrosine-HRP detection antibody, and then detected with an enhanced Chemi reagent A and Chemi reagent B. To analyze the phosphorylated proteins, the relative expression of specific phosphorylated proteins was determined, following a quantification of scanned images by Image J.

### Human Phospho-Kinase assay

Human Phospho-Kinase assay kit was also purchased from R&D Systems. Before extracting the protein, PANC-1 cells were treated with 5 μM Crizotinib. The membranes were incubated with cell lysates overnight at 4°C and washed with washing buffer 3 times. To analyze the phosphorylated proteins, a cocktail of biotinylated detection antibodies, streptavidin-horseradish peroxidase and chemiluminescent detection reagents were used. The relative expression of specific phosphorylated proteins was also analyzed following a quantification of scanned images by Image J.

### Matrigel plug assay

Animal care and experimental procedures were conducted in accordance with the Guide for Animal Experiments by the Korean Academy of Medical Sciences. Male, 6-week old BALB/c mice were obtained from the ORIENT-BIO Laboratory Animal Research Center (Gyeonggi-do, Korea). The animals were fed with standard mice chow with free access to tap water in a 21°C and humidity controlled animal house with alternating 12 hr light-dark cycles. The mice were subcutaneously injected with 700 μl of Matrigel (CORNING, Tewksbury, MA) containing concentrated VEGF (50 ng/mL) and either Crizotinib (5 μM) or PBS. After 7 days, mice were sacrificed and the Matrigel plugs were fixed in 4% buffered formaldehyde, which was embedded in paraffin, and sectioned. The 8 μm-thick sections were stained with hematoxylin and eosin (H&E). H&E staining was performed to identify the formation and infiltration of new, functional microvessles. Functional vessels with intact RBSs were quantified manually using a microscope [high-powerfield (HPF) x200].

### Tumor xenograft study

To establish PANC-1 tumors in mice, PANC-1 pancreatic cancer cells were grown in culture, then detached by trypsinization, washed, and resuspended in PBS. Six weeks old athymic BALB/c nude mice were injected with 5×10^6^ cells in the right flank of each mouse to initiate tumor growth. When the tumor volume reached to 50~100 mm^3^, mice were randomly divided into two groups, each having five mice. Mice were fed with Crizotinib at a dose of 50 mg/kg by oral gavage, 6 times a week for 33 days, respectively. For the control group, 0.2 mL vehicle was fed. The body weight and tumor size were recorded every three days. The tumor size was calculated by 0.5 × long axis × (short axis)^2^.

### Immunohistochemistry

After deparaffinization, immunostaining was performed using 8 μm-thick sections of the tumor samples. Microwave antigen retrieval was performed in a citrate buffer (pH 6.0) for 5 min prior to peroxidase quenching with 3% hydrogen peroxide (H_2_O_2_) in PBS for 10 min. The sections were then washed in water and preblocked with a normal goat or horse serum for 1 hr. Next, the tissue sections were incubated overnight at 4°C in 1:50 dilutions of anti-PCNA (abcam), cleaved caspase-3 (Cell Signaling Technologies), and p-ALK (Tyr^1604^; abcam). After washing the sections with PBS, they were incubated with biotinylated secondary antibodies (1:100) for 1 hr and then streptodavidin-HRP was applied. Finally, the sections were developed with diaminobenzidine tetrahydrochloride substrate for 10 min, and counterstained with hematoxylin. At least three random fields of each section were examined at a magnification of 400 x.

### Statistical analysis

Data were expressed as the mean ± S.D. A statistical analysis was performed using ANOVA. A P-value of 0.05 or less was considered statistically significant. Statistical calculations were performed using SPSS software for Windows operating system (Version 10.0; SPSS, Chicago, IL).
